# New cellular tools reveal complex epithelial–mesenchymal interactions in hepatocarcinogenesis

**DOI:** 10.1038/sj.bjc.6604440

**Published:** 2008-07-01

**Authors:** S Sagmeister, M Eisenbauer, C Pirker, T Mohr, K Holzmann, H Zwickl, C Bichler, D Kandioler, F Wrba, W Mikulits, C Gerner, M Shehata, O Majdic, B Streubel, W Berger, M Micksche, K Zatloukal, R Schulte-Hermann, B Grasl-Kraupp

**Affiliations:** 1 Department of Medicine I, Division: Institute of Cancer Research, Medical University of Vienna, Borschkegasse 8a, Vienna A-1090, Austria; 2 Department for Surgery, Medical University of Vienna, Währinger Gürtel 18-20, Vienna A-1090, Austria; 3 Institute for Clinical Pathology, Medical University of Vienna, Währinger Gürtel 18-20, Vienna A-1090, Austria; 4 Department of Medicine I, Division for Hematology, Medical University of Vienna, Währinger Gürtel 18-20, Vienna A-1090, Austria; 5 Institute of Immunology, Medical University of Vienna, Borschkegasse 8a, Vienna A-1090, Austria; 6 Department of Pathology, Medical University of Graz, Auenbruggerplatz 25, Graz A-8036, Austria

**Keywords:** hepatocarcinogenesis, tumour stroma, epithelial–mesenchymal interactions

## Abstract

To enable detailed analyses of cell interactions in tumour development, new epithelial and mesenchymal cell lines were established from human hepatocellular carcinoma by spontaneous outgrowth in culture. We obtained several hepatocarcinoma (HCC)-, B-lymphoblastoid (BLC)-, and myofibroblastoid (MF)-lines from seven cases. In-depth characterisation included cell kinetics, genotype, tumourigenicity, expression of cell-type specific markers, and proteome patterns. Many functions of the cells of origin were found to be preserved. We studied the impact of the mesenchymal lines on hepatocarcinogenesis by *in vitro* assays. BLC- and MF-supernatants strongly increased the DNA replication of premalignant hepatocytes. The stimulation by MF-lines was mainly attributed to HGF secretion. In HCC-cells, MF-supernatant had only minor effects on cell growth but enhanced migration. MF-lines also stimulated neoangiogenesis through vEGF release. BLC-supernatant dramatically induced death of HCC-cells, which could be largely abrogated by preincubating the supernatant with TNF*β*-antiserum. Thus, the new cell lines reveal stage-specific stimulatory and inhibitory interactions between mesenchymal and epithelial tumour cells. In conclusion, the new cell lines provide unique tools to analyse essential components of the complex interplay between the microenvironment and the developing liver cancer, and to identify factors affecting proliferation, migration and death of tumour cells, neoangiogenesis, and outgrowth of additional malignancy.

Hepatocellular carcinomas are devastating cancers with increasing worldwide incidence and mortality rates (Parkin *et al*, 2005). The challenges posed by these often lethal cancers are daunting, with conventional treatment options being limited. Chronic hepatitis due to unresolved viral infections, excessive intake of ethanol, or metabolic disorders has been identified as major risk factors (Llovet *et al*, 2003; Farazi and DePinho, 2006). The viral infections and other noxes usually result in cell damage, which evokes inflammatory responses. The resulting chronic hepatitis and associated fibrosis and cirrhosis are the major driving force for the development of malignant liver cells (Ramadori and Saile, 2004; Bataller and Brenner, 2005; Robinson and Coussens, 2005; Drucker *et al*, 2006).

The inflammatory response of the liver involves activation of immune and other mesenchymal cells, such as endothelial and stellate cells (Ramadori and Saile, 2004; Steiling *et al*, 2004; Tuchweber *et al*, 2006). The latter ones play a leading role in the development of fibrosis following their transition to myofibroblasts, which synthesise the main components of the extracellular matrix. These different cell types release a plethora of growth factors and proinflammatory mediators including cytokines and reactive oxygen species, many of which are involved in growth regulation of the epithelial cell compartment of the tissue. If release of these signals is unbalanced and prolonged, selective growth of (pre)malignant cells in the tissue may occur. This accelerates tumour promotion and progression (Robinson and Coussens, 2005; Drucker *et al*, 2006). Therefore, the microenvironment appears as a primary factor in determining whether dysfunctional epithelial cells will continue to grow or not.

The gradual formation of liver cancer is accompanied by the development of a specific tumour microenvironment, which is composed of immune cells, small vessels, myofibroblastic cells, and extracellular matrix components (Faouzi *et al*, 1999; Bhowmick *et al*, 2004; Albini and Sporn, 2007). Tumour-associated myofibroblasts are a rich source of extracellular matrix-degrading proteases and of cytokines. They are, therefore, capable of remodelling connective tissue, stimulate invasion, migration, and growth of tumour cells, and induce neoangiogenesis (Dubuisson *et al*, 2000; Monvoisin *et al*, 2002). Furthermore, important cellular components of the tumour microenvironment are various types of leukocytes, including dendritic cells, natural killer cells, and tumour-associated macrophages (Robinson and Coussens, 2005; De Visser *et al*, 2006; Whiteside, 2006). Their interaction with tumour cells often results in immunotolerance through reduced immunogenicitiy of the tumour and incapacitation of immune cells. The immune cells may even increase the potential of cancer cells to progress, proliferate, and metastasise, that is, the tumour-associated macrophages release metalloproteinases for local tissue destruction and interleukin (IL)-6 and IL-8 for endothelial cell invasion into the tumour (De Visser *et al*, 2006; Whiteside, 2006). In summary, there is strong evidence that the various mesenchymal cell types in the (pre)malignant tissue undergo a multiplicity of deviations, crucial for cancer formation, progression, and maintenance.

However, the precise role of specific mesenchymal cells and secreted factors during the stepwise development of liver cancer is still largely unknown. Detailed pertinent studies are hampered by the paucity of well-defined human cell lines. The most commonly used hepatic epithelial lines are HepG2 and Hep3B deriving from human hepatoblastoma and hepatocellular carcinoma, WRL68 from a human embryonal liver, and CCL13 Chang liver cells, with no available information on the source and with reported contamination by Hela-cells (www.atc.org). As the lines have been isolated decades ago, a huge number of diverse subclones exist that notoriously produce heterogeneous and sometimes conflicting data. Hardly any information is available on some of the more recently established hepatocellular carcinoma lines (Saito *et al*, 1989; Park *et al*, 1995; Li *et al*, 2003; Hu *et al*, 2004). A few hepatic mesenchymal lines from human liver or liver tumours have been isolated. Similar to the existing epithelial cells, these mesenchymal lines appear to be barely authenticated, ill characterised and standardised (Arthur 1996; Xu *et al*, 2005).

In this study, epithelial and two types of mesenchymal cell lines were reproducibly isolated from human hepatocarcinomas (HCCs). Mesenchymal cells were identified as B-lymphoblastoid (BLC)- and myofibroblastoid (MF)-cells, reflecting two of the key cellular constituents of the microenvironment in liver tumours. Extensive characterisation revealed that these new lines have retained many biological and functional characteristics of the cells of origin. We found that all of the mesenchymal cell lines dramatically enhance growth of liver cancer prestages in an *in vitro* model. This may partially explain the fact that HCCs frequently arise multifocally (Llovet *et al*, 2003; Farazi and DePinho, 2006). In advanced stages, MF-cells stimulate cell migration and neoangiogenesis. Interestingly, the BLC-lines induce death of HCC-cells, apparently through TNF*β* secretion. This observation may be of interest from a therapeutic point of view. In conclusion, the new cell lines reveal stage-specific stimulatory and inhibitory interactions between mesenchymal and epithelial tumour cells. They offer new tools to unravel the mechanistic role of the microenvironment during hepatocarcinogenesis.

## Materials and methods

### Establishment of cell lines

Patients with primary liver tumours were subjected to surgical resection. A fully documented patient's history and informed consent were obtained in each case (see also online Supplementary material). The study protocol conforms to the ethical guidelines of the 1975 Declaration of Helsinki, as reflected by the approval of the ‘Ethic Committee of the Vienna Medical University’. Tumour samples were fixed and processed as described (Raidl *et al*, 2004). Additional samples were transferred into transport medium (RPMI1640; Sigma, St Louis, MO) containing 10% FCS (PAA, Linz, Austria), 100 U ml^−1^ penicillin-streptomycin (PAA), 2.5 *μ*g ml^−1^ fungizone (Sigma) and 100 *μ*g ml^−1^ gentamycin (Biochrom AG, Berlin, FRG). The establishment of cell lines followed published protocols (Frazier *et al*, 1990; Park *et al*, 1995). In brief, tumour tissue was cut into pieces of approximately 0.5 mm^3^, which were incubated in ACL4-medium and 5% FCS at 37°C and 5% CO_2_. Outgrowing semi-adherent cells were separated and cultivated. Fibroblasts disappeared after approximately 13 weeks, were overgrown by tumour cells, or eliminated by differential trypsinization. Established cell lines were maintained in RPMI1640 and 10% FCS (HCC- and BLC-lines) or ACL4-medium and 5% FCS (MF-lines). Cells were regularly tested by the Mycoplasma Detection Kit (Roche, Mannheim, FRG).

### Treatment with conditioned supernatant

Control media: standard medium of indicator cells (50% v/v) and unconditioned medium routinely used for the cells tested (50% v/v). Treatment: standard medium of indicator cells (50% v/v) and medium conditioned for 72 h by the cells tested (50% v/v). Immunoneutralisation: medium or conditioned supernatants were pre-incubated with neutralising antibodies (see online Supplementary material) at 37°C for 60 min.

### Tumourigenicity

Using three animals per cell line, 2 × 10^6^ cells in 100 *μ*l of 0.9% NaCl were subcutaneously injected into SCID/BALB/c mice. Tumour formation was viewed periodically by palpation (Gotzmann *et al*, 2002). Formalin-fixed and paraffin-embedded sections of xenocrafts were stained with hematoxylin–eosin. The experiments were approved by the ‘Committee of Animal Protection of the Austrian Ministry of Sciences’ and performed according to Austrian regulations, which agree with the criteria outlined in the ‘UKCCCR Guidelines for the Welfare of Animals in Experimental Neoplasia’ in 1998.

Karyotyping, fluorescence *in situ* hybridisation (FISH) and comparative genomic hybridisation (CGH) of the cell lines were performed as described previously (Raidl *et al*, 2004; Streubel *et al*, 2006).

### Sequencing

Exons 2 and 3 of Ras-protoncogenes were sequenced, as given elsewhere (Macheiner *et al*, 2006). For the primers applied see Supplementary material.

## Telomeric repeat amplification protocol-assay

Telomeric repeat amplification protocol-assay followed the recent descriptions (Kim and Wu, 1997). Amplification products were separated by PAGE and visualised by FluorImager-595 (Molecular-Dynamics, Sunnyvale, CA, USA). One total product generated (TPG) corresponds to 600 molecules of telomere substrate primers extended by at least four telomeric repeats within 30 min at 30°C. TPGs were calculated per cell, based on protein amount and cell numbers applied. Data were derived from 2–4 experiments.

### Immunodetection

For primary antibodies and ELISA-kits used see Supplementary material. For immunohistology, cells were processed as described recently (Gotzmann *et al*, 2002; Drucker *et al*, 2006). Immunoreactions were visualised by application of cye-dye-conjugated secondary antibodies and TCS-SP confocal microscopy (Leica, Heidelberg, FRG). Detailed discription of FACS analyses of the semi-adherent cell lines and antisera applied are given elsewhere (Pfistershammer *et al*, 2004; Bluml *et al*, 2005). ELISA-kits were applied according to the manufacturers' instructions.

### Testing for growth factors in primary hepatocyte cultures

SPF Wistar rats, obtained from and kept at the ‘Division for Decentralized Biomedical Facilities Vienna’, received 250 mg of *N*-nitrosomorpholine per kilogram body weight by gavage to induce the formation of preneoplastic hepatocytes. Twenty-one days later livers were perfused with collagenase. The cell suspension obtained was purified from mesenchymal cells and seeded on collagen-coated Petri dishes. For further details see Drucker *et al*, 2006.

### DNA content and DNA replication

For autoradiography newly synthesised DNA was labelled with ^3^H-thymidine (60–80 Ci mmol^−1^; ARC, St Louis, MO), which was added at 0.5 *μ*Ci ml^−1^ medium 24 h before harvesting. For further processing see Drucker *et al*, 2006.

### RT−PCR

Total RNA was extracted and transcribed, as described (Drucker *et al*, 2006). For primers used see Supplementary material. PCR products were separated in 1.2% agarose gels, stained with ethidium bromide, and visualised by UV-light. Viral DNA of Epstein-Barr-virus (EBV) was detected as given elsewhere (Bauer *et al*, 2005; Drucker *et al*, 2006).

### Proteomics

Cells were incubated with ^35^S-methionine for 6 h for metabolic labelling of proteins synthesised within this period. The cell supernatant (secretome) was processed and subjected to two-dimensional (2D) gel electrophoresis and subsequent autoradiography. Fluorescent intensities of cytosolic protein spots on 2D-gels were normalised to the intensities of *β*-actin to determine relative amounts. Spots were selected for protein identification by mass spectrometry. For details see Zwickl *et al*, 2005.

## Human umbilical vein endothelial cells

Human umbilical vein endothelial cells (HUVEC) were isolated from umbilical cords by approved protocols (Jaffe *et al*, 1973) and kept on fibronectin-coated plates (Chemicon, Hampshire, UK) in M199-medium with 20% FCS (GibcoBRL, Grand Island, NY) and 10 *μ*g of endothelial cell growth supplement (ECGS, Upstate-Biotechnology, NY) per ml at 37°C in 5% CO_2_. Cells were used after 3–6 passages.

## Results

### Establishment and characterisation of cell lines

**Figure 1:**
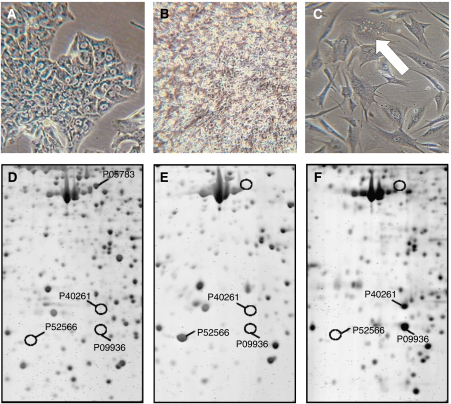
Morphology and proteome pattern differing in hepatocarcinoma, B-lymphoblastoid, and myofibroblastoid cell-lines. Light microscopy of HCC-1.2 in (**A**), BLC-1 in (**B**), and MF-2 cells in (**C**). Arrow in (**C**) indicates lipid droplets. Magnification: × 80. In (**D**–**F**) cytosolic proteins of the lines were separated by 2D-PAGE and detected by fluorography. Selected proteins were further identified by mass spectrometry (Zwickl *et al*, 2005). A segment of a representative 2D-PAGE gives highly different protein profiles in (**D**) HCC-1.2, in (**E**) BLC-2, and in (**F**) MF-2 cells. Swiss Prot numbers: P05783, keratin type 1, cytoskeletal 18; P09936, ubiquitin carboxyl-terminal hydrolase isozyme L1; P40261, nicotinamide N-methyltransferase; and P52566, rho GDP-dissociation inhibitor 2.

Specimens from six hepatocellular carcinomas and one undifferentiated primary liver tumour were transferred to culture. Generally, outgrowth of big, semiadherent aggregates occurred after approximately 7 weeks (Figure 1B). About 6 weeks later, cells appeared exhibiting epithelial (Figure 1A) or myofibroblastoid-like morphology (Figure 1C).

#### Epithelial cells (HCC-lines)

**Figure 2:**
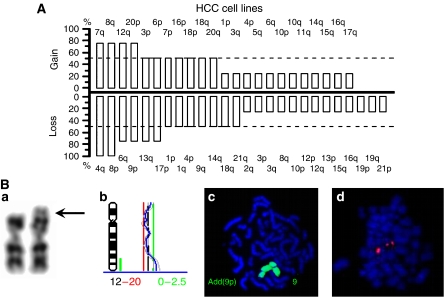
Genomic alterations in hepatocarcinoma and B-lymphoblastoid cells. In (**A**) relative frequencies of chromosomal alterations in HCC-1.1, HCC-1.2, HCC-2, and HCC-3, as analysed by CGH. Columns give percentage of lines with gains or losses of DNA on the chromosomal arms indicated. In (**B**) cytogenetic analyses of BLC-4: (**a**) G-banding reveals elongation of 9p (arrow); (**b**) blue line indicates the green to red fluorescence ratio profile of chromosome 12. The grey lines give the 95% confidence interval. Green bar besides the chromosome ideogram indicates a gain on 12q (arrow). (**c**) FISH for chromosome 9 (green) shows that the additional material on 9p is chromosome 9-negative. (**d**) FISH for the immunoglobin heavy chain region demonstrates normal signals on chromosome 14.

Many functions of the cells of origin were found to be preserved. We determined deviations from the normal genotype by CGH. Frequency and pattern of alterations in the lines highly resembled those recently found in human hepatocellular carcinoma (Figure 2; Raidl *et al*, 2004). Gains of DNA clustered in chromosome arms 7q, 8q, 12q, and 20p and losses were often found at 4q, 6q, 8p, 9p, and 13q. We also sequenced the hotspots of Ki-Ras, Ha-Ras, and N-Ras. No mutations could be found in any of the four lines, which reflects a further characteristic feature of liver cancer (Llovet *et al*, 2003; Stahl *et al*, 2005; Farazi and DePinho, 2006). Despite of multiple genetic alterations in the CGH analysis the tumourigenicity of the lines was weak. Two of the cell lines formed tumours in one of three inoculated mice only (Table 1).

The HCC-lines were positive for the epithelial marker proteins cytokeratin 8 and 18 and expressed the glycolysis enzyme lactate dehydrogenase, which is a feature common to most cultured tumour cells (Figure 1). To study the secretome of the HCC-lines we applied a novel proteomics approach, which is based on metabolic labelling of secreted proteins (Zwickl *et al*, 2005). All cell lines release hepatocyte-specific proteins, such as albumin, *α*1-antitrypsin, serotransferrin, and apolipoprotein A1 and E to the medium. Furthermore, they secrete a complement cytolysis inhibitor, a characteristics of tumour cells. The level of *α*-fetoprotein in the secretome was low in three of the four lines investigated, which reflects the features of the corresponding liver tumour (Table 1 and online Supplementary material).

A hallmark of hepatocytes is their versatility to bioactivate diverse toxic chemicals. Accordingly, the cell lines express phase-I (cytochrome-P450 1A1, 1B1, 3A4, and 2E1) and phase-II enzymes (glutathione-S-transferase and sulfotransferase 1A1) at levels similar to human liver (data not shown). Enzymes coping with reactive oxygen species, such as superoxide dismutases and catalase, were expressed as well (Table 1).

Detailed comparison of the genotype and the expression of liver-specific proteins of the new HCC-lines and of the already established HepG2- and Hep3B-cells are given as online Supplementary material. The new lines appear to have retained more features of hepatocytes than the already established cell lines.

#### Semi-adherent cells (BLC-lines)

These lines contained parts of the EBV genome at a mean load of approximately 25 copies per cell. Considering the genomic alterations detected by the cytogenetic methods, they revealed much less deviations than the HCC-cell lines. The line BLC-4 revealed gains of 12q24.1-ter and an elongated 9p, indicating that additional genetic material from 12q24.1-ter was translocated to the short arm of chromosome 9 (Figure 2B). BLC-7 harboured additional chromosomes 9, 12, and 14. The karyotypes of the two further cell lines showed no aberrations. FISH for the chromosomal regions coding for immunoglobulin heavy chains (14q32) revealed no translocation in all cells studied. Furthermore, lines were found to be monoclonal as determined by the rearrangement pattern in the IgG-locus (Table 1). In SCID mice all cell lines tested (BLC-1, -2, and -4) produced anaplastic tumours exhibiting vast areas of necrosis and frequent mitosis.

The BLC-lines revealed a lymphoblastoid phenotype with many features of activated B-cells, as examined by FACS analyses (Table 1). The cells also displayed some characteristics of activated T-cells, which may be due to their infection with EBV. FITC-labelled latex beads were taken up by the majority of the cells. Upon stimulation with PMA or LPS they released superoxide to the medium. Thus, the cell lines highly resemble B-cells, and exhibited some features known from macrophages such as phagocytosis and superoxide-production (Table 1; Parzefall *et al*, 2001; Teufelhofer *et al*, 2003).

#### Myofibroblastoid cells (MF-lines)

These cell lines are characterised by a population doubling time of more than 150 h and a barely detectable telomerase activity (Table 1). They exhibited minor genetic changes. Loss of genetic material in 4q and 6p was evident in two of the lines investigated (MF-2 and MF-6). No tumourigenicity in SCID mice was observed with any of the MF-lines.

The cell lines expressed considerable amounts of various microfilaments, such as *α*-smooth muscle actin, fibulin-2, tenascin, and vimentin and, therefore, meet the main characteristics of myofibroblasts. The expression of the junctional proteins plakoglobin and N-cadherin, as observed in the MF-lines, has been recently described in hepatic stellate cells after their activation to myofibroblasts and in stellate cell lines (Vogel *et al*, 2000; Proell *et al*, 2005; Xu *et al*, 2005). Their proteome pattern differed largely from that of the HCC- and BLC-cells (Table 1; Figure 1).

### Interactions between epithelial and mesenchymal cells in early and advanced stages of hepatocarcinogenesis

#### B-lymphoblastoid and myofibroblastoid cells stimulate growth of early stages of hepatocarcinogenesis

**Figure 3:**
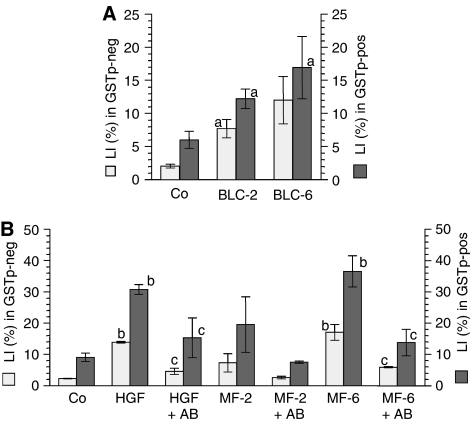
B-lymphoblastoid and myofibroblastoid cells release growth factors for unaltered (GSTp-neg) and premalignant (GSTp-pos) hepatocytes. Treatment of hepatocytes commenced 4 h after seeding and lasted for 68 h. Abbreviations of treatment groups: (Co), control medium; (BLC-2) or (BLC-6), medium supernatant conditioned by BLC-2 or BLC-6; (MF-2) or (MF-6), medium supernatant conditioned by MF-2 or MF-6 cells; (HGF), aliquots of a HGF stock solution (Sigma-Aldrich; 20 *μ*g ml^−1^ PBS/0.1% BSA) were added for finally 20 ng ml^−1^ medium; (HGF+AB), (MF-2+AB), or (MF-6+AB), HGF-containing medium or conditioned supernatants were pre-incubated with anti-HGF. In (**A**) and (**B**): ^3^H-thymidine was added 24 h before harvesting. DNA synthesis was determined by autoradiography. In each experiment 2000 nuclei of GSTp-neg cells and 600 nuclei of GSTp-pos cells were evaluated per treatment group. The labelling index (LI%) was calculated as percentage of labelled hepatocyte nuclei per total number of hepatocyte nuclei counted. Columns: LI (%) of replicating GSTp-neg (□) and GSTp-pos (▪) hepatocytes. Means±s.e.m. of at least three separate liver cell preparations are given. Statistics by Kruskal–Wallis test: Co *vs* cell supernatant or HGF: (**a**) *P*<0.05; (**b**) *P*<0.01; cell supernatant *vs* neutralised supernatant or HGF *vs* neutralised HGF: (**c**) *P*<0.05.

To study the impact of mesenchymal cells on the development of liver tumours, we applied a recently developed *ex vivo* culture model as a screening tool (Drucker *et al*, 2006). Due to the lack of reliable markers and culture models for human liver cancer prestages this system uses premalignant rat hepatocytes, which are detectable by their selective expression of placental glutathione-S-transferase (GSTp-pos). GSTp-pos cells show an approximately fourfold higher probability for DNA replication than unaltered (GSTp-neg) cells indicating an inherent growth advantage, the main characteristic of precancerous cells (Figure 3). Incubation with the supernatant of BLC- or MF-cells raised DNA replication and DNA content of primary hepatocytes (Figure 3). When discriminating between unaltered and premalignant hepatocytes, the highest induction of DNA synthesis occurred in the premalignant, GSTp-pos cells by supernatant of MF-6, followed by supernatant of MF-2, BLC-6, and BLC-2. Fourty percent of GSTp-pos hepatocytes were stimulated by factors released by MF-6 to replicate DNA, which is remarkably high for hepatocytes in primary culture. The effect of MF-2 and MF-6 supernatant could be largely abrogated by pre-incubation with neutralising anti-HGF (Figure 3B), whereas anti-HGF was without effect on the BLC-supernatant (data not shown). Thus, HGF appears to be the main growth stimulator released by the MF-cells, whereas the growth-inducing component in the BLC-supernatants remains to be identified. In conclusion, the factors released by the two different mesenchymal cell types appear to stimulate preferential outgrowth of tumour prestages.

#### B-lymphoblastoid cells induce death of hepatocarcinoma cells

**Figure 4:**
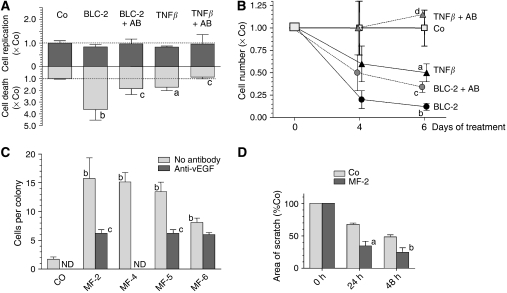
B-lymphoblastoid cells induce death of hepatocarcinoma cells whereas myofibroblastoid cells enhance neoangiogenesis and migration of hepatocarcinoma cells. In (**A**) and (**B**): HCC-2 cells were treated 24 and 72 h after seeding and were harvested after 4 and 6 days. Abbreviations of treatment groups: (Co), untreated HCC-2; (TNF*β*), aliquots of a TNF*β* stock (Sigma-Aldrich; 1 *μ*g ml^−1^ PBS/0.1% BSA) were added for finally 1.5 ng ml^−1^ medium; (BLC-2), HCC-2 exposed to medium supernatant conditioned by BLC-2; (TNF*β*+AB) or (BLC-2+AB), TNF*β*-containing medium or conditioned supernatant were pre-incubated with anti-TNF*β*. In (**A**), cells were kept for 96 h. ^3^H-thymidine was added 24 h before harvesting, and DNA replication was determined by autoradiography. To assay apoptosis by FACS-analyses, cells were incubated in 0.5 ml PBS containing 15 *μ*g propidium iodide (Sigma-Aldrich) for 30 min at 4°C and were analysed in a Becton-Dickinson FACSCalibur system. In (**B**) cells were harvested and counted. In (**A**) and (**B**) means±s.d. from three separate experiments are given. Statistics by Kruskal–Wallis test; Co *vs* cell supernatant or TNF*β*: (**a**) *P*<0.05; (**b**) *P*<0.01; cell supernatant *vs* neutralised supernatant or TNF*β vs* neutralised TNF*β*: (**c**) *P*<0.05; (**d**) *P*<0.01. In (**C**) HUVEC were seeded at 1 × 10^3^ per cm^2^. After cell attachment supplements in M199-medium were reduced to 1% FCS and no ECGS for 24 h before start of treatment. Abbreviations of treatment groups: (Co), control medium; (MF-2), (MF-4), (MF-5), or (MF-6), medium supernatant conditioned by the MF-cells. Control media or conditioned supernatants were pre-incubated with anti-vEGF. Treatments were renewed after 72 h for further 96 h. The size of the HUVEC colonies was determined by counting the number of cells. Experiments were performed in triplicate and at least 10 colonies per well were scored. Abbreviations: ND, not done. In (**D**) confluent HCC-2 cultures were scratched manually with a 200 *μ*l pipette tip, followed by rinsing and treatments. Abbreviations of treatment groups: (Co), control medium; (MF-2), medium supernatant conditioned by MF-2 cells. Total area of the scratches was measured by morphometry (Lucia 6.0, Nikon, Düsseldorf, FRG). In (**C**) and (**D**) mean±s.e.m. of at least three independent studies are given. Statistics by Kruskal–Wallis test; Co *vs* cell supernatant: (**a**) *P*<0.05; (**b**) *P*<0.01; cell supernatant *vs* neutralised supernatant: (**c**) *P*<0.05.

The HCC-2, BLC-2, and MF-2 cell lines were all isolated from the same donor and were, therefore, chosen to study interactions between the different cell types in liver cancer. Supernatant of MF-2 had minor effects on number, replication, and death of HCC-2 and also of other hepatocarcinoma cell lines (data not shown). However, BLC-2 supernatant induced dramatically cell death in HCC-2 cultures (Figure 4A). In parallel, a slightly reduced fraction of cells in S-phase was evident (Figure 4A). Accordingly, after 4 days of treatment cell numbers were only approximately 15% of controls (Figure 4B). A similar effect was also seen when treating HCC-1.2, HCC-3, or HepG2 cells with supernatant conditioned by BLC-1, -4 and -7 (data not shown).

The BLC lines secrete considerable amounts of TNF*β* (Table 1). The cytocidal effect of the cells could be simulated by treating HCC-2 with TNF*β* at a concentration being equivalent to that in the BLC-supernatant. When TNF*β*-containing medium or BLC-2 supernatant were preincubated with neutralising TNF*β*-antiserum, the effect of recombinant TNF*β* was blocked completely and of the BLC-2 supernatant incompletely (Figure 4A and 4B). This indicates that additional factors may be involved in the tumouricidal effects of the BLC cells.

#### Myofibroblastoid cells produce angiogenic factors and increase migration of hepatocarcinoma cells

We hypothesised that supernatants from MF-lines could stimulate tumour progression by enhancing neoangiogenesis and migration of hepatocarcinoma cells. In fact, MF-cells produce angiogenic factors, as reflected by an increased size of HUVEC colonies (Figure 4C). Antibodies neutralising vascular endothelial growth factor (vEGF) largely blocked colony growth of the endothelial cells indicating that vEGF is the main angiogenic factor secreted by the lines. Also HCC-cells release considerable amounts of vEGF, as determined by the HUVEC-assay (data not shown) and by ELISA (Table 1).

To determine the effects of MF-cells on the migratory capability of HCC-cells, we performed scratch assays (Figure 4D). Medium conditioned by MF-2 cells significantly accelerated scratch closure rates in HCC-2 cultures when compared to control medium. Similar results were obtained when treating HCC-1.2 lines with MF-6 derived supernatant (data not shown). This indicates that MF-cells increase considerably the ability of hepatocarcinoma cells for migration.

## Discussion

**Figure 5:**
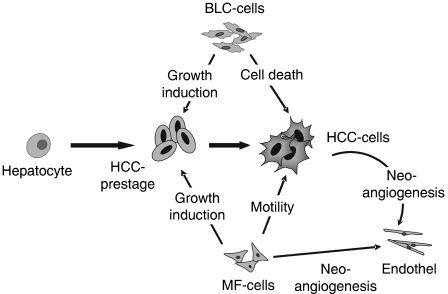
Complex interactions of host tissue with developing liver cancer. Hepatocarcinogenesis is characterised by multiple steps of increasing aberrations in cellular signalling networks, which starts in the transition from normal cells to early precursor lesions. The gradual formation of liver cancer is accompanied by the development of a specific tumour microenvironment, consisting of immune cells, small vessels, myofibroblasts, and extracellular matrix components. In advanced stages of tumour development, myofibroblastoid (MF)-cells enhance migration of hepatocarcinoma (HCC)-cells and neoangiogenesis. In contrast, the B-lymphoblastoid (BLC)-cells induce death of the malignant hepatocytes. Furthermore, BLC- and MF-cells release factors that stimulate growth of premalignant hepatocytes. Thus, the complex interactions between the microenvironment and the different stages of epithelial transformation affect proliferation, migration and death of cells, neoangiogenesis, and outgrowth of additional liver tumours.

The present work describes the establishment of several hepatocellular, B-lymphoblastoid and myofibroblastoid cell lines from human hepatocellular carcinoma and provides mechanistic explanations for their complex interactions at different stages of hepatocarcinogenesis (Figure 5).

Each of the three types of cell line represents a distinct cellular identity, as determined by advanced techniques. CGH revealed that the epithelial HCC-cells show a profile of genomic alterations, which differs clearly from that of the BLC- and the MF-lines. We also studied the proteome and applied a novel method to discriminate between intracellular and secreted proteins (Zwickl *et al*, 2005). We found that all line types studied exhibit a characteristic and cell-type specific proteome and secretome pattern. Furthermore, most of the HCC-cell lines have maintained features characteristic for hepatocytes and secrete albumin, serotransferrin, and the *γ*-chain of fibrinogen. The proteome pattern also revealed considerable expression of various enzymes metabolising drugs, aldehydes or reactive oxygen species (Table 1). Accordingly, the HCC lines have been shown to bioactivate diverse genotoxic compounds, such as polycyclic hydrocarbons, aflatoxin B1, and nitrosamines (Winter *et al*, submitted). Thus, the new HCC-cell lines exhibit a capability for drug-metabolism similar to the tissue of origin.

The BLC-lines highly resemble B-cells, exhibit also some features known from macrophages, and contain several copies of the EBV-genome per cell. All our donors harboured a chronic, subclinical EBV-infection, according to a rate of approximately 95% EBV-carriers in the European population (Herrmann and Niedobitek, 2003). The virus immortalises the infected cells to proliferate indefinitely in culture most probably by inducing autocrine loops of TNF*α*, TNF*β*, and IL-10 (Rochford *et al*, 1997). Our EBV-positive BLC-cells produce high levels of TNF*β*, IL-6, and TGF*β*1 and variable levels of TNF*α*. In addition, two of the four lines appeared transformed with gross karyotypic abnormalities (Figure 2). It is presently unclear whether these genomic aberrations were caused by the EBV in the donor or occurred during establishment of the lines *in vitro*.

Hepatocellular carcinoma were reported to recruit and activate hepatic stellate cells or portal fibroblasts to tumour-associated myofibroblasts (Faouzi *et al*, 1999). The transdifferentiation of fibroblasts into myofibroblasts is modulated by cancer cell-derived cytokines, such as TGF*β* (Bieri and Moses, 2006). The MF-lines established from the hepatocellular carcinoma meet many characteristics of hepatic stellate cells activated to tumour-associated myofibroblasts. They express *α*-smooth muscle actin, fibulin-2, vimentin, and the junctional proteins plakoglobin and N-cadherin as recently described for activated stellate stells (Vogel *et al*, 2000; Monvoisin *et al*, 2002; Steiling *et al*, 2004; Proell *et al*, 2005; Tuchweber *et al*, 2006). Furthermore, the MF-cells contain lipid droplets, which highly resemble the cytoplasmic storage site for retinyl ester in stellate cells (Figure 1C). All these features together suggest that the MF-lines rather derive from stellate cells than from portal fibroblasts.

Cell–cell interactions have a major impact on carcinogenesis in the liver and other organs (Albini and Sporn, 2007). Along these lines, we recently found that DNA synthesis of premalignant hepatocytes is increased by growth factors released from Kupffer and endothelial cells of normal liver (Drucker *et al*, 2006). The present work shows that cancer-derived mesenchymal cells also stimulate growth of premalignant hepatocytes (Figure 5). The new BLC cell lines produce TNF*α*, TNF*β*, TGF*β*1, IL-6, and presumably IL-10 (Table 1; Rochford *et al*, 1997). In recent studies, TNF*α* and IL-6 had no impact on DNA replication of premalignant hepatocytes, whereas TGF*β*1 even suppressed the growth (Löw-Baselli *et al*, 2000; Drucker *et al*, 2006). In this work, 0.7 or 1 ng of TNF*β* ml^−1^ medium lowered DNA-replication of hepatocytes in primary culture by 57 and 76%, respectively (data not shown). IL-10 has been described to exert anti-inflammatory and cytoprotective effects in the liver and to exert minor effects on hepatocyte replication (Louis *et al*, 1998). Thus, the factors responsible for the pronounced growth stimulation of the first stages of hepatocarcinogenesis by the BLC-lines remain to be identified.

It is controversially discussed whether the latent EBV infection accelerates the onset of HCC by supporting HCV replication, exacerbating the inflammatory processes in the liver, or by direct growth stimulation of the tumour cells (Sugawara *et al*, 1999; Herrmann and Niedobitek, 2003). In any case, tumour-associated immune cells are often localised close to the border of the tumour. It, therefore, appears likely that the cytokines released by the BLC-cells act on both, the tumour itself and on the tumour prestages in the vicinity (Ramadori and Saile, 2004; Drucker *et al*, 2006). Thus, the EBV infection may stimulate B-cells to promote carcinogenesis by enhancing outgrowth of the tumour prestages. This may contribute to the fact that HCCs often arise multifocally, which complicates therapy and worsens prognosis.

In contrast to the effects on premalignant hepatocytes, BLC-supernatant exerted a pronounced tumouricidal effect in the HCC-cells (Figure 5), and other cell lines tested, such as HepG2- Hep3B-, MCF7-, and CRL2020-cells (data not shown). One candidate cytokine is TNF*β*, which is secreted by all our BLC-lines. As a prototypical member of the TNF-superfamily, it induces death of a wide range of tumour cells, which is based on activation of the immune system and on direct killing of tumour cells expressing the appropriate TNF-receptors (Strand *et al*, 1996; Tamada and Chen, 2006). Under our conditions recombinant TNF*β* simulates partly the effects of the BLC-supernatant implying that additional factors may be involved. TNF*α* has sequence homologies to TNF*β* and exerts similar biological properties (Tamada and Chen, 2006). However, only two of the BLC-lines expressed this cytokine, although all lines are capable of inducing cell death. Further examples for tumouricidal effects through members of the TNF-superfamily are the cd40/cd40l(cd154)- and the cd95/cd95l-systems (Strand *et al*, 1996; Tamada and Chen, 2006). However, our BLC-cells were positive for cd40 but negative for cd40l and cd95l, indicating that these death-inducing systems are not operative in the BLC-supernatant. Although being detrimental to tumour cells, the EBV-DNA load in hepatocellular carcinoma was found to be 1000-fold higher than in peripheral blood mononuclear cells (Sugawara *et al*, 2000; Tamada and Chen, 2006). It appears possible that the tumour controls and inactivates these cells, as described for other infiltrating leukocytes (De Visser *et al*, 2006; Whiteside, 2006).

Myofibroblasts are a rich source of growth factors, including TGF*β*1, platelet-derived growth factor, FGF2, FGF7, HGF, and others (Monvoisin *et al*, 2002; Ramadori and Saile, 2004; Steiling *et al*, 2004; Bataller and Brenner, 2005). We recently found that the addition of FGF7 or HGF to the medium dramatically raises replication of premalignant hepatocytes, which may explain the growth stimulatory effect of the MF-supernatant on the GSTp-pos hepatocytes and the abrogation of this effect by neutralising anti-HGF antibodies in this study (Drucker *et al*, 2006; Abb.3). Thus, this cell type may also contribute to the outgrowth of additional malignancy within the liver. In advanced stages of hepatocarcinogenesis, the effects of the MF-cells appear to be different from growth stimulation (Figure 5). They synthesise considerable amounts of vEGF for neoangiogenesis and increase the motility of the HCC-cells (Figure 4). We could not obtain any evidence regarding the fact that the increased migration of the HCC-lines by the MF-derived supernatant was due to HGF or FGF7 (data not shown).

In conclusion, the immune cells and myofibroblasts, derived from hepatocellular carcinoma, release a plethora of cytokines that act on both, the tumour itself and on tumour prestages. Thus, the new cell lines described in the present work are a unique tool to unravel the complex functional interplay of epithelial cells and the microenvironment at different stages of liver cancer development.

## Figures and Tables

**Table 1:** Characterisation of cell lines

**Epithelial cell lines (HCC-1.1, HCC-1.2, HCC-2, and HCC-3)**			
***Population doubling time***	37.3–46 h	***Detoxification*** * Cytochromes P4501A1, 1B1, 3A4, and 2E1, Sulfotransferase 1A1*^b^^,^^c^^,^^h^^,^^j^	++
***Telomerase activity* a**	18–65 TPG	*Aldehyde dehydrogenase Liver carboxylesterase 1, Glutathione-S-transferase* b	+
***Tumourigenicity***	+/−	***Antioxidation*** * CuZn Superoxide dismutase, Mn Superoxide dismutase*^b^	++
***Epithelial/Hepatocyte markers*** *Albumin, α1-Antitrypsin, Fibrinogen γ-chain, Apolipoprotein E, Cytokeratin 18*^b^^,^^j^^,^^k^	++	*Thioredoxin, Catalase* b ^,^ c	+
*Cytokeratin 8, Fatty acid binding protein liver-type, Serotransferrin, Apolipoprotein A1, Haptoglobin*^b^^,^^j^^,^^k^	+	***Other proteins*** * Lactat dehydrogenase, Complement cytolysis inhibitor*^b^	++
*α-Fetoprotein*^b^	+/−	*Vitamin D binding protein, Enoyl-CoA hydratase* b	+
***Secretion of cytokines*** *IL-1β, IL-4, IL-5, TNFα, TNFβ*^d^	−	***Neoangiogenesis*** * Secretion of vEGF (∼1000)*^d^	++
			
**Non-adherent cell lines (BLC-1, BLC-2, BLC-4, BLC-6, and BLC-7)**			
***Population doubling time***	19–23 h	***Myeloic cell markers*** * CD13, CD14, CD15, CD16, CD25, CD33 CD36, CD38, CD64, Myeloperoxidase*^e^	−
***Telomerase activity* a**	6.1−16 TPG	***Superoxide production* f**	++
***Tumourigenicity***	++	***Phagocytosis*** * Uptake of latex beads*^f^	+
***Leukocyte marker*** *CD45*^e^	++	*Phagocytic glycoprotein I (CD44), MHCI, MHCII* e	+
***B-cell marker*** C*D19*^e^	++	***Secretion of cytokines*** * IL-6 (∼3000), IFNα (∼70), TGFβ1 (∼1000), TNFα (∼200),TNFβ (∼1000)* d	++
***Progenitor B-cell marker*** *CD10*^e^	−	*IL-1β, IL-4, IL-5* d	−
***Activated B-cell markers*** *CD23, B7H1*^e^	++	***TNF/TNFR-system*** * CD40* e	+
***B-CLL marker*** *CD5*^e^	−	*CD40L* b *, CD95 L (FAS-L)* e ^,^ d	−
***T-cell markers*** *CD1a, CD3, CD4, CD8*^e^	−	***Further proteins*** * Rho GDP-dissociation inhibitor 2*^b^^,^^k^^,^^l^	+
***T-lymphocyte activation antigens*** *CD80, CD86*^e^	+	*Hematopoietic lineage cell-specific protein, Coronin 1a, Gelsolin, Coactosin, I-Plastin* b ^,^ l	+
***T-cell surface glycoprotein E2*** *CD99*^e^	+	***Monoclonality* i**	+
***Dendritic cell markers*** *Langerin, bdca3, bdca4*^e^	−		
			
**Myofibroblastoid cell lines (MF-1, MF-2, MF-3, MF-5, and MF-6)**			
***Population doubling time***	181–267 h	***Epithelial markers*** * Cytokeratin 7 and 8 (Cam 5.2) Cytokeratin 8 and 18*^g^	−
***Telomerase activity* a**	<2 TPG	***Endothelial markers*** *ICAM1 (cd54), Von Willebrand Factor, PECAM1 (cd31)* g	−
***Comparative genomic hybridisation***	Loss in 4q, 6p, 13q	***Growth factors*** * Hepatocyte growth factor, Keratinocyte growth factor*^c^	+
***Tumourigenicity***	−		−
***Myofibroblast markers*** *Tenascin*^c^	++	***Further proteins*** * Integrin α2, Integrin αV, Matrix metalloproteinase 14, Transgelin*^b^	+
*α-Smooth muscle actin, Fibulin-2 Vimentin* c ^,^ g	++	*Gelsolin, Cofilin, Ezrin* b ^,^ l	+
*Plakoglobin, N-Cadherin* g	+	*Nicotinamide N-methyltransferase, Ubiquitin carboxyl-terminal hydrolase isozyme L1, Collagen α type 1,2, and 3; Fibronectin* b ^,^ j ^,^ l	+
		***Secretion of cytokines*** * IL-1β, IL-4, IL-5, TNFα, TNFβ*^d^	−

Data are given if at least 2 of the cell lines were investigated and if all of the lines investigated showed identical features. (−), negative; (+/−), weakly positive; (+), positive; (++), strongly positive.

a Telomerase activity gives the mean TPG unit per cell. Determined by:

b 2D PAGE/MS.

c RT−PCR.

d ELISA; number in parentheses gives concentration of cytokine in pg ml^−1^ medium.

e FACS analyses according to (Pfistershammer *et al*, 2004; Bluml *et al*, 2005).

f Methods described in (Parzefall *et al*, 2001; Teufelhofer *et al*, 2003).

g Immunoflourescence.

h Quantitative RT−PCR.

i Characterisation of the rearrangement pattern of the IgG locus, as described in (Wan *et al*, 1990). Not expressed in:

j BLC-cells.

k MF-cells.

l HCC-cells.
