# Supplementary material for: New cellular tools reveal complex epithelial–mesenchymal interactions in hepatocarcinogenesis
Source: Br J Cancer. 2008 Jul 1;99(1):151–9. doi: 10.1038/sj.bjc.6604440 (PMC2453035; doi:10.1038/sj.bjc.6604440)
Supplement: Supplementary material [file 6604440x1.doc]

**Online supplemental material**

*Table. Age and sex of the patients, causative factors for disease,* *and grade (HCC/1-HCC/3) an*d *pTNM stage of the hepatocellular carcinoma.* Abbreviations: m, male; f, female; HCV, hepatitis-C-virus infection.

| **Age** | **Sex** | **Etiology** | **Diagnosis** | **α-Fetoprotein (kU/L)** | **Cell lines derived** |
| --- | --- | --- | --- | --- | --- |
| 56 | m | HCV | HCC/2, pT1, pN0, pM0 | 2,2 ^a)^ | HCC-1.1., HCC1.2, BLC-1, MF-1 |
| 55 | m | unknown | HCC/3, pT3, pNx, pM0 | 4,8 | HCC-2, BLC-2, MF-2 |
| 50 | m | HCV | HCC/2, pT1, pN0, pM0 | 343 | HCC-3, MF-3 |
| 79 | m | unknown | Undifferentiated primary liver carcinoma | 2,7 | BLC-4 |
| 69 | f | HCV | HCC/1, pT1, pNx, pMx, | < 2 | MF-5^b)^ |
| 43 | w | unknown | HCC/2, pT4, pNx, pMx | 379 | MF-6, BLC-6 |
| 78 | w | HCV | HCC/3, pT2, pN1, pMx | 66,2 | BLC-7 |

a) physiological range of AFP: 0-7 IU/ml serum.

b) derives from a HCC-metastasis to the subcutis.

Table. Primers used for RT-PCR and sequencing

| ***Primer*** | ***Sequence*** |
| --- | --- |
| **Catalase** sense  antisense | 5´-CCACTGTTGCTGGAGAATCG-3´ 5´-CCGGAT CCTTCAGATGTGTC-3´ |
| **CYP1A1** sense  antisense | 5′-TCCAGAGACAACAGGTAAAACA-3′ 5′-AGGAAGGGCAG AGGAATGTGAT-3′ |
| **CYP2E1** sense  antisense | 5´-TAATACGACTCACTATAGGACAGGGACAGGGGAATCAT-3´  5´-TGGGGTCCAGAGATTGATGCAGGCAAGTAGTGTAG AAAG-3´ |
| **Fibulin 2 sense  antisense** | **5´-CACCACGGAGAGTTTCAGAG-3´ 5´-GCAGTGAGAAGCCAGGAAAG-3´** |
| **Hepatocyte Growth Factor sense  antisense** | **5´-CAGAGGGACAAAGGAAAAGAA-3´ 5´-GCAAGTGAATGGAAGTCCTTTA-3** |
| **Keratinocyte Growth Factor sense  antisense** | **5´-GAACAAGGAAGGAAAACTCTATGCAA-3´ 5´-AAGTGGGCTGTTTTTTGTTCTTTCT-3´** |
| **α-Smooth muscle actin sense  antisense** | **5´-CGTGGCTATTCCTTCGTTAC-3´ 5´-TGCCAGCAGACTCCATCC-3´** |
| **Sulfotransferase 1A1 sense  antisense** | **5´-GCAACGCAAAGGATGTGGCA-3´ 5´-**TCCGTAGGACACTTCTCCGA-3´ |
| **Tenascin sense  antisense** | **5´-GAGAAAGGCAGACACAAGAG-3´ 5´-GCAGTCCAGTTGAGTTTGAG-3´** |
| **Vimentin sense  antisense** | **5´-ccagatgcgtgaaatggaag-3´ 5´-TGAGTGGGTATCAACCAGAG-3´** |
| **Ha-Ras/Exon 1 sense  antisense** | 5´-GAGACCCTGTAGGAGGACCC-3´ 5´-GG GTGCTGAGACGAGGGACT-3´ |
| **Ha-Ras/Exon 2 sense  antisense** | 5´-AGAGGCTGGCTGTGTGAACT-3´ 5´-CAT GGCATTAGCAAAGAC-3´ |
| **Ki-Ras/Exon 1 sense  antisense** | 5´-ACTGAATATAAACTTGTGGTAGTTGGACCT-3’ 5´-TCAAAGAATGGTCCTGGACC-3´ |
| **Ki-Ras/Exon 2 sense  antisense** | 5´-TCAAGTCCTTTGCCCATTTT-3´ 5´-TGCATGGCATTAGCAAAGAC-3´ |
| **N-Ras/Exon 1 sense  antisense** | 5´-GAACCAAATGGAAGGTCACA-3´  5´-TGGGTAAAGATGATCCGACA-3´ |
| **N-Ras/Exon 2 sense  antisense** | 5´-TCTTACAGAAAACAAGTGGT-3´ 5´-GTAGAGGTTAATATCCGCAA-3´ |

***Table. Source of antibodies ad kits applied.***

| **Immunofluorescence:** |  |
| --- | --- |
| anti-cytokeratin 7 and 8 (CAM 5.2) | Becton Dickinson, San Jose, CA |
| anti-cytokeratin 8 | Dakocytomation, Glostrup, Denmark |
| anti-cytokeratin 18 | Dakocytomation, Glostrup, Denmark |
| anti-fibulin-2 | kind gift of Dr.Timpl, Max-Plank Institute, Martinsried, Germany) |
| anti-α-smooth muscle actin | Dakocytomation, Glostrup, Denmark |
| anti-vimentin | Dakocytomation, Glostrup, Denmark |
| anti-Van Willebrand factor | Dakocytomation, Glostrup, Denmark |
| anti-ICAM-1 | Dakocytomation, Glostrup, Denmark |
| anti-plakoglobin | Transduction Laboratories, Lexington, UK |
| anti-N-cadherin | Transduction Laboratories, Lexington, UK |
| anti PECAM (cd31, | Pharmingen Bioscience, Heidelberg, FRG |
| anti-rat Yp subunit of placental glutathione-S-transferase | Biotrin-International, Dublin, Eire |
| **Immunoneutralization:** |  |
| anti-HGF (**0.1 µg per ml medium)** | R&D Systems, Minneapolis, MN |
| anti-vEGF (**1µg per ml medium)** | **R&D Systems, Minneapolis, MN,** |
| **anti-TNFß (7.5 µg per ml medium)** | Sigma-Aldrich, St. Loius. MO |
| **ELISA-kits:** |  |
| interferon 2 | Bender MedSystems, Vienna, Austria |
| transforming growth factor ß1 | Bender MedSystems, Vienna, Austria |
| transforming growth factor 2 | Bender MedSystems, Vienna, Austria |
| tumor necrosis factor α | Bender MedSystems, Vienna, Austria |
| tumor necrosis factor ß | Bender MedSystems, Vienna, Austria |
| interleukin 1ß | Bender MedSystems, Vienna, Austria |
| interleukin 4 | Bender MedSystems, Vienna, Austria |
| interleukin 5 | Bender MedSystems, Vienna, Austria |
| vascular endothelial growth factor | R&D Systems, Minneapolis, MN |
| **Assays for quantitative RT-PCR** |  |
| cytochrome P450 1A1 | Applied Biosystems, Foster City, USA |
| cytochrome P450 1B1 | Applied Biosystems, Foster City, USA |
| cytochrome P450 3A4 | Applied Biosystems, Foster City, USA |

***Table. Comparison of HCC-cell lines to Hep3B- and Hep2-cells.* Abbreviations: (-), negative; (+), weakly positive; +, positive; ++, strongly positive.**

|  | ***HCC-1.1, HCC-1.2 HCC-2, HCC-3^a)^*** | ***Hep3B  (ATCC-No 8064)^b)^*** | ***HepG2 (ATCC-No 8065)^b)^*** |
| --- | --- | --- | --- |
| ***Population doubling time*** | 37.3 – 46 hrs | ~25 hrs | ~ 41 hrs |
| ***Telomerase activity ^c)^*** | 18 – 65 TPG | 87 TPG | 20 TPG |
| ***Comparative genomic hybridization*** | **Gains in:** 3p,6p,7p,7q 8q,12q,16p,18p,18q, 20p,20q; **Losses in**: 1p 1q,4p,4q,6q,8p,9p,9q, 13q,14q,17p,18q,21q | **Gains in**: 5p,5q,7p,9q 15q,16p,16q,17p,20q, **Losses in:** 1p,2q,3p,3q 4p,4q,6q,9p,11q,12q 13q,14q,17q,18p,18q,20p | **Gains in**: 14q,17q,20q  **Losses in**: 1p,3p,4p,4q 5p,5q,9p,9q,10p,10q, 11p,12q,18p,18q,21q |
| ***Tumorigenicity*** | (+) | ***+*** *^b)^* | - *^b)^* |
| ***Epithelial/Hepatocyte markers*** *Albumin ^d)^* | ++ | *-* | ++ |
| *α1-Antitrypsin ^d)^* | ++ | - | + |
| *Fibrinogen γ-chain ^d)^* | ++ | - | + |
| *Apolipoprotein E ^d)^* | ++ | - | (+) |
| *Apolipoprotein A1  ^d)^* | + | - | (+) |
| *Cytokeratin 18 ^d)^* | ++ | **(+)** | + |
| *Serotransferrin ^d)^* | + | - | + |
| ***Tumormarker*** *α-Fetoprotein ^d)^* | (+) | - | + |
| ***Neoangiogenesis*** *Secretion of VEGF (~1000) ^f)^* | ++ | n.d. | + |
| ***Detoxification*** *Cytochromes P4501A1 ^d,e,g)^* | ++ | n.d. | - |
| *Cytochrome P450 1B1 ^g)^* | ++ | n.d. | + |
| *Cytochromes P4502E  ^d,e)^* | ++ | n.d. | - |
| *Cytochrome P450 3A 4 ^g)^* | ++ | n.d. | (+) |
| *Sulfotransferase 1A 1 ^d,e)^* | ++ | n.d. | (+) |
| *Aldehyde dehydrogenase ^d)^* | + | - | + |
| *Placentar Glutathione-S-  transferase ^d)^* | + | - | (+) |
| ***Antioxidation*** *CuZn Superoxiddismutase ^d)^* | ++ | (+) | + |
| *Mn Superoxiddismutase ^d)^* | ++ | - | (+) |
| *Thioredoxin  ^d)^* | + | - | + |
| *Catalase ^d,e)^* | + | + | (+) |
| ***Other proteins*** *Lactat dehydrogenase ^d)^* | ++ | +++ | ++ |
| *Vitamin D binding protein,^d)^* | + | - | (+) |
| *Enoyl-CoA hydratase ^d)^* | ++ | - | ++ |

a) Data are given if at least 2 of the 4 cell lines were investigated and if all of the lines investigated showed identical features; b) for details see: <http://www.atcc.org/>; c) Telomerase activity gives the mean TPG unit + SD per cell.

Determined by: d) 2D PAGE/MS; e) RT-PCR; f) ELISA; number in parentheses gives concentration of cytokine in pg/ml medium; g) quantitative RT-PCR.
